# Design of a three-segment continuum robot for minimally invasive surgery

**DOI:** 10.1186/s40638-016-0035-1

**Published:** 2016-03-24

**Authors:** Bo Ouyang, Yunhui Liu, Dong Sun

**Affiliations:** Department of Mechanical and Biomedical Engineering, City University of Hong Kong, Kowloon, Hong Kong China; Department of Mechanical and Automation Engineering, The Chinese University of Hong Kong, Shatin, China

**Keywords:** Continuum robot, Dimensional synthesis, Visual servo, Medical robot

## Abstract

Continuum robot, as known as snake-like robot, usually does not include rigid links and has the ability to reach into a confined space by shaping itself into smooth curves. This paper presents the design of a three-segment continuum robot for minimally invasive surgery. The continuum robot employs a single super-elastic nitinol rod as the backbone and concentric disks assembled on the backbone for tendons attachment. Each segment is driven by four tendons and controlled by two linear actuators. The length of each segment is optimized based on the surgical workspace. A visual servo system is designed to assist the surgeon in operating the robot. Simulation experiment is conducted to demonstrate the proposed design.

## Background

A continuum robot is a flexible robot inspired by caterpillars, elephant trunks, octopus arms, and mammalian tongues. The robot can vary its nature shape because of the materials flexibility and is capable of reaching into a complex environment. Therefore, the continuum robot has the potential in single-port access surgery and natural orifice transluminal.

**Fig. 1:**
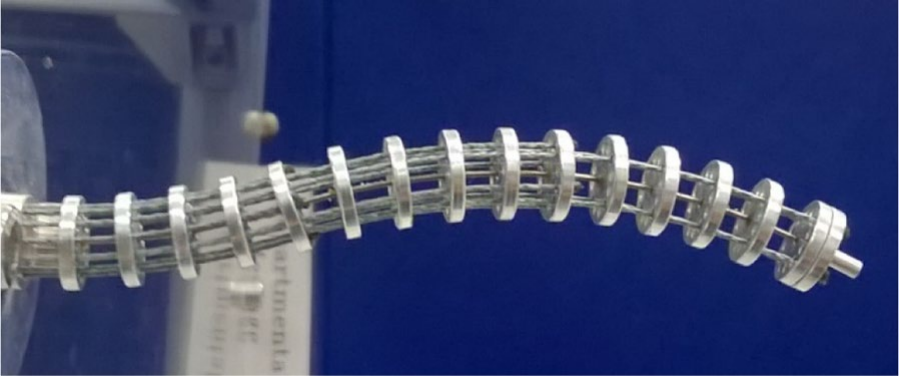
A three-segment continuum robot prototype. It is shaped by twelve tendons connected to six linear actuators

The basic elements of a continuum robot are backbone, actuators, and disks, as shown in Fig. 1. An elephant trunk-like multi-segment continuum robot has been designed by using tendons as the actuators [1]. The multi-backbones continuum robots have been developed for the surgeries in throat and abdomen [2, 3]. This robot has a primary backbone, and other backbones were regarded as the actuators. Active catheter is another type of continuum robot, which employs the tube as the backbone [4].

It is generally assumed that the segment of continuum robot bends with constant curvature [5]. The kinematics of multi-segment continuum robot can be formulated by a Denavit–Hartenberg-type approach [1]. Although there are various ways for kinematic modeling, the piecewise constant curvature is assumed finally [6]. Variable curvature continuum robot has been also developed [7]. However, the kinematic modeling is extremely hard.

Control of continuum robot possesses a great challenge because of the compliance of continuum robot. The dynamic model of a planar continuum robot has been introduced [8]. The dynamics of a spatial continuum robot has also been reported based on the principle of virtual power [9]. The statics and dynamics of variable curvature continuum robot have been presented by the classical Cosserat rod model [10]. However, the design of a controller is still a difficult issue because of the material flexibility. A neural network controller has been tried, where a hypothesis dynamic model is estimated online [11]. A model-less feedback control has been proposed without using the constant curvature kinematic frameworks [12].

A three-segment continuum robot for minimally invasive surgery has been developed in this study. The robot employs a single super-elastic nitinol rod as the backbone. Twelve tendons passing through the concentric disks are used to operate the robot. These tendons are divided into three groups. Each group has two pairs of tendons and is controlled by two linear actuators. The segment length is determined by the cable nodes in the group, which can be adjusted by varying the position of the nodes. Moreover, the approximate boundary of reachable workspace is formulated. A unique method is proposed to minimize the length of continuum robot. Finally, a visual servo system is designed.

## Mechanical structure

**Fig. 2:**
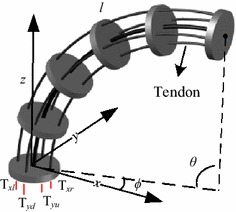
One segment of continuum robot. The mechanical structure of a continuum robot usually contains tendons, disks, and backbone

The mechanical structure of the proposed continuum robot is shown in Fig. 2. The backbone material is a super-elastic rod. The robot is shaped by tendons passing through the disks. One segment of the continuum robot has two degrees of freedom (DOFs), i.e., rotation around *z*-axis ($\phi$) and *y*-axis ($\theta$), based on the constant curvature kinematics frameworks. The number of tendons is at least three for driving one segment, because tendons must be work in tension. This property brings a disadvantage for the kinematic control of continuum robot with three tendons. Because the shape of one segment is determined by two tendons, the tension of the third tendon is extremely large as the translation error of the third tendon is positive. The third tendon would be snapped. The designed continuum robot with each segment driven by four tendons and controlled by two linear actuators is developed to compensate this disadvantage.

**Fig. 3:**
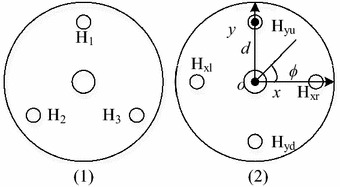
Disk in each segment. (1) The disk of continuum robot driven by three tendons; (2) the disk of continuum robot driven by four tendons

One of the differences between the continuum robot developed in this paper and the continuum robots driven by three tendons is arrangement of actuators. Two common arrangements of actuators are shown in Fig. 3. The second one is selected to arrange the tendons, because the two pairs ($H_{x}$ and $H_{y}$) of tendons are uncoupled with each other. Here, linear motor is selected because it can provide large range of motion. Furthermore, each pair of tendons is connected to a single linear motor. One segment of the continuum robot is driven by six motors. The manufacturing cost is thus lower. The question is whether it is possible to steer four tendons by two linear actuators only. This question will be answered by addressing the kinematic model of continuum robot.

The configuration of each segment robot is defined by three parameters: the curvature ($k\left( {\varvec{\uprho}} \right)$), the angle of the plane containing the arc ($\phi \left( {\varvec{\uprho}} \right)$), and arc length ($l$), as shown in Fig. 2, where ${\varvec{\uprho}}$ is the tendon length and $k\left( {\varvec{\uprho}} \right) = \theta /l$. By comparing the varied length of tendons in each pair, one can find

1 $$\Delta l = 2\left( {l - 2n\frac{l}{\theta }\sin \left( {\frac{\theta }{2n}} \right)} \right) \ge 0,$$

where *n* is the number of disks. Equation (1) indicates that one tendon works in tension and the other one is slack in each pair, which is exactly the requirement of the continuum robot control. Therefore, one just needs to change the moving direction of linear motor based on $\phi$ for the operation of the continuum robot. On the other side, multiple segments are employed to provide sufficient DOF for accomplishing complex surgical tasks. Twelve tendons and six linear motors are used to operate the robot finally. The tendons are divided into three groups ($H_{1}$, $H_{2}$, and $H_{3}$), and each group has two pairs ($H_{ix}$, $H_{iy}$, and $i = 1, 2, 3$). The length of each segment ($l_{i}$) is defined by the cable nodes. Thus, the length of each segment can be adjusted based on the surgical requirement.

### Dimensional synthesis

The three-segment continuum robot developed in this paper has six DOFs. The workspace of each segment is determined by three parameters: $\phi_{i}$, $\theta_{i}$, and $l_{i}$, $i = 1,2,3$. The range of rotation angle $\phi_{i}$ is 0°–360°. Note that the rotation angle $\theta_{i}$ is limited because of the constant curvature kinematics. In general, the range of rotation angle $\theta_{i}$ is ranged 0°–90° or 0°–120°. The entire attitude space is independent of the arc length $l_{i}$. However, the position of end effector depends on $l_{i}$. The arc length of each segment should be optimized based on surgical workspace.

In this paper, the range of $\theta_{i}$ is set as 0°–90°. The reachable workspace of the continuum robot is a circular symmetry in geometry, so it can be defined by the cross section in plane. The approximate boundary of the cross section can be formulated. For the three-segment continuum robot, its left approximate boundary of the cross section can be divided into four sections:

2 $$s_{1} = {\mathbf{T}}_{1} \left( {\phi_{1} ,\theta_{1} } \right)\left[ {0 0 l_{2} + l_{3} 1} \right]^{\text{T}} ,$$

3 $$s_{2} = {\mathbf{T}}_{1} \left( {\phi_{1} ,\theta_{1} } \right){\mathbf{T}}_{2} \left( {\phi_{2} ,\frac{ - \pi }{2}} \right){\mathbf{T}}_{3} \left( {\phi_{3} ,\frac{ - \pi }{2}} \right)\left[ {0 0 0 1} \right]^{\text{T}} ,$$

4 $$s_{3} = {\mathbf{T}}_{1} \left( {\phi_{1} , \frac{ - \pi }{2}} \right){\mathbf{T}}_{2} \left( {\phi_{2} ,\theta_{2} } \right)\left[ {0 0 l_{3} 1} \right]^{\text{T}} ,$$

5 $$s_{4} = {\mathbf{T}}_{1} \left( {\phi_{1} , \frac{ - \pi }{2}} \right){\mathbf{T}}_{2} \left( {\phi_{2} ,\frac{ - \pi }{2}} \right){\mathbf{T}}_{3} \left( {\phi_{3} ,\theta_{3} } \right)\left[ {0 0 0 1} \right]^{\text{T}} ,$$

where $\phi_{1} ,\phi_{2} ,\phi_{3} = 0$, $\theta_{1} ,\theta_{2} ,\theta_{3} \in \left( {0, \pi /2} \right]$, ${\mathbf{T}}_{i}$ is the transform matrix of the $i$th segment, and $x \le 0$. If $\phi_{1}$ changes from 0 to $2\pi$, the cure of each section turns into a surface, which forms the approximate boundary of workspace.

Now the dimensional synthesis of continuum robot can be analyzed based on the requirement of surgical workspace. Suppose that the surgical workspace is a cuboid, i.e., $x \in \left[ { - x_{r} ,x_{r} } \right]$, $y \in \left[ { - y_{r} ,y_{r} } \right]$ and ${\text{z}} \in \left[ {z_{rd} ,z_{ru} } \right]$, and $x_{r} \ge y_{r}$. The case of $x_{r} \le y_{r}$ is similar, because the workspace is circular symmetry. The cross section of surgical workspace is a rectangle in the $xz$ plane. Denote the vertexes as ${\mathbf{V}}_{j}$, $j = 1, \ldots ,4$, and the middle point of ${\mathbf{V}}_{3} \mathbf{V}_{4}$ as ${\mathbf{V}}_{0}$. Based on the geometric property of boundary, the cuboid is in the workspace of continuum robot by providing that ${\mathbf{V}}_{0}$, ${\mathbf{V}}_{1}$, …, ${\mathbf{V}}_{4}$ are all in the workspace. If the robot length is very long, the robot will easily go out of the channel with a small rotation angle *θ*. Therefore, the length should be minimized to improve the dexterity of the continuum robot. The dimensional synthesis can be described as the following optimization problem:

6 $$\begin{array}{*{20}l} { \hbox{min} } \hfill & {l_{1} + l_{2} + l_{3} } \hfill \\ {{\text{s}} . {\text{t}} .} \hfill & {\left\{ {\begin{array}{*{20}l} {T\left( {\phi_{1j} ,\theta_{1j} ,l_{1} , \ldots ,\phi_{3j} ,\theta_{3j} ,l_{3} } \right){\mathbf{d}}^{e} = {\mathbf{V}}_{j} , \quad j = 0, \ldots ,4} \hfill \\ { - \frac{\pi }{2} \le \theta_{ij} \le \frac{\pi }{2}, \quad l_{i} > 0,\quad i = 1,2,3, \quad j = 0, \ldots ,4} \hfill \\ \end{array} } \right.} \hfill \\ \end{array}$$

where ${\mathbf{T}} = {\mathbf{T}}_{1} {\mathbf{T}}_{2} {\mathbf{T}}_{3}$ is the transformation from the base coordinate frame $F_{\text{b}}$ to the frame $F_{\text{e}}$ of the end effector, and ${\mathbf{d}}^{e}$ represents the end point of the end effector in the frame $F_{\text{e}}$. Therefore, the length of each segment can be determined through optimization.

## Visual servo system design

After the mechanical structure of continuum robot is developed, the next step is to design an interactive system for assisting surgeon in controlling the robot in a user-friendly way.

It is assumed that camera can capture all the feature points on each segment of the continuum robot. Then, the configuration of segment can be determined by the feature point (${\mathbf{F}}_{i}$) on the top disk. Based on the projection principle, the image coordinate of feature point is

7 $$z_{1}^{c} \left[ {\mathbf{m}_{1} /f\varvec{ }1} \right]^{T} = {\mathbf{R}}_{ce} {\mathbf{R}}_{1} {\mathbf{F}}_{1} + {\mathbf{R}}_{ce} {\mathbf{P}}_{1} + {\mathbf{p}}_{ce} .$$

where $\mathbf{m}_{1}$ is the image coordinate of feature point, $f$ is the focal length, $z^{c}$ is an arbitrary scale factor, and ${\mathbf{R}}_{ce}$ and ${\mathbf{p}}_{ce}$ are the extrinsic parameters of camera, and ${\mathbf{R}}_{1}$ and ${\mathbf{P}}_{1}$ are the rotation matrix and translation of the first segment, respectively. After eliminating $z_{1}^{c}$, a nonlinear equation is obtained:

8 $$\mathbf{m}_{1} \varvec{ } = \varvec{ }{\mathbf{g}}\left( {\phi_{1} , \theta_{1} } \right) ,$$

The numerical solution of configuration ($\phi_{1} ,\theta_{1}$) is calculated based on Eq. (8). The configuration of the end effector can be determined based on kinematics. On the other side, Eq. (8) can be applied to calculate the image Jacobian matrix

9 $$\left[ {\begin{array}{*{20}c} {{\text{d}}u_{1} } & {{\text{d}}v_{1} } \\ \end{array} } \right]^{T} = {\mathbf{J}}_{1}^{ } \left( {\phi_{1} , \theta_{1} } \right)\left[ {\begin{array}{*{20}c} {{\text{d}}\phi_{1} } & {{\text{d}}\theta_{1} } \\ \end{array} } \right]^{T} .$$

where $u_{1}$ and $v_{1}$ are the coordinate in pixel. The image Jacobian matrix of the last two segments can also be derived by the above steps. The Jacobian matrix of the continuum robot can be calculated based on the kinematics. Then, one can design a controller based on Eq. (9).

### Simulation experiment

**Fig. 4:**
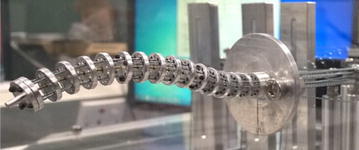
The three-segment continuum robot prototype. It is driven by 12 cables and 6 linear actuators

To verify the design of the continuum robot, the continuum robot prototype was developed. The system consists of linear actuators, cables, pulleys, drivers, camera, digital pen, and digitizer tablet, as shown in Fig. 4. The super-elastic nitinol rod was employed as the backbone of the continuum robot. Twelve cables passed through disks around the backbone. These cables were divided into three groups with respect to three segments. Each group has two pairs of cables, and each pair of cables is connected to a linear actuator. So each segment of continuum robot is just driven by two motors. Furthermore, each segment length can be varied by adjusting cable nodes in each group based on task requirement.

**Fig. 5:**
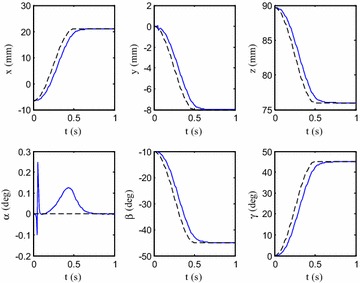
Response of the control system. *Solid line* and *dotted line* represent the desire configuration and the response, respectively

To control the continuum robot, a PD controller was designed with visual feedback. The desired configuration was set as $$y_{\text{d}} = \left[ {\begin{array}{*{20}c} {21} & { - 8} & {76} & {0^{\circ} } & { - 45^{\circ} } & {45^{\circ} } \\ \end{array} } \right]$$. The following function was employed for motion planning:

10 $$y = \left\{ {\begin{array}{*{20}l} {y_{\text{s}} + \frac{{\left( {y_{\text{d}} - y_{\text{s}} } \right)}}{2}({ \sin }\left( {\frac{\pi t}{{t_{\text{d}} }} - \frac{\pi }{2}} \right) + 1)} \hfill & {t \in \left[ {0, t_{\text{d}} } \right]} \hfill \\ {y_{\text{d}} } \hfill & {t \in \left( {t_{\text{d}} , \infty } \right]} \hfill \\ \end{array} } \right..$$

where $y_{\text{s}}$ is the initial pose. Here, proportional gain $K_{\text{P}}$ was set as 1.5, and differential gain $K_{\text{d}}$ was selected as 0.1. The response of the control system in simulation is shown in Fig. 5. One can find that the error converged to zero although the response had a little time delay.

## Conclusion

This paper presents the design of a three-segment continuum robot. The approximate boundary of workspace is formulated. The configuration of each segment is determined. In the future, the visual servo control system will be developed, and the controller will be improved.
